# CalOPT: A Specialty Pharmacy–Dietitian Quality Improvement Initiative for Calcium Optimization in Patients with Osteoporosis Risk

**DOI:** 10.3390/pharmacy13040100

**Published:** 2025-07-23

**Authors:** Jennifer Cerulli, Alisha Roberts, Ellie Wilson, Scott Guisinger

**Affiliations:** 1 Department of Pharmacy Practice, Albany College of Pharmacy and Health Sciences, Albany, NY 12208, USA; 2 Northeast Shared Services, Schenectady, NY 12308, USAsguisinger@northeastsharedservices.com (S.G.)

**Keywords:** osteoporosis, calcium supplementation, specialty pharmacy, patient education

## Abstract

A total of 38% of Americans do not meet the Recommended Dietary Allowance (RDA) for calcium including those at risk for osteoporosis. To increase the percentage of patients at risk for osteoporosis who achieve goal calcium RDA intake, a collaborative specialty pharmacy-registered dietitian-nutritionist (RDN) quality improvement program was developed. Patients aged 18 to 90 years old receiving osteoporosis therapy (denosumab, teriparatide, zoledronic acid) or medications that increase bone loss (elagolix, oral prednisone) were provided with a structured assessment and educational intervention. Daily calcium intake included patient self-reported dietary intake plus supplement use. Written and verbal education on increasing dietary intake based on patient preferences was provided with 5 calcium-rich food-source store coupons. Recommendations for supplement selection (citrate vs. carbonate) and/or medication-related problem resolution were provided. Follow-up occurred at 3–6 months. Fifty patients enrolled [94% female, mean age 66.6 years (SD 15.3)] were taking denosumab (36), teriparatide (1), zoledronic acid (1), elagolix (7) and prednisone (5). The mean baseline daily dietary calcium intake was 500 mg (SD 247) with none achieving goal intake with diet alone. Average calcium supplement use in 22 (44%) patients was 686 mg daily (SD 284). At baseline, 17 (34%) met goal daily calcium intake compared to 30 (60%) at post intervention follow-up (*p* = 0.009). Over half of the store coupons were redeemed. A specialty pharmacy-RDN customized intervention program provides a model for aiding patients to modify calcium intake.

## 1. Introduction

Osteoporosis is a significant public health concern due to the morbidity and mortality associated with fractures in addition to the associated humanistic and socio-economic outcomes incurred [1,2,3]. Despite the availability of pharmacologic treatments to reduce fracture risk, gaps in care remain. The osteoporosis treatment or “care gap” is the difference between the number of individuals who would benefit from osteoporosis management and the number receiving optimal care [3,4]. To close the gap, interventions are primarily focused on osteoporosis detection and the initiation of prescription pharmacotherapy to reduce fractures. As numerous factors contribute to the gap, a multifaceted approach to osteoporosis management delivered by physician and non-physician health care professionals (HCP) which incorporates patients in shared decision making is warranted [3,4,5,6]. Patient outcomes are improved by HCP engagement in various stages of the osteoporosis care continuum including risk identification and screening, medication initiation, adherence optimization, and lifestyle modification counseling [3,6,7,8].

Despite the variable evidence regarding the role calcium and vitamin D therapy play in reducing fracture risk, there is consensus that HCP should offer interventions to include nutrient education and counseling as part of osteoporosis care [2,3,5,6]. United States (US) national guidelines recommend that adults must consume 1000 to 1200 mg of calcium in their diet daily, with supplementation, when necessary, for the prevention and treatment of osteoporosis [2,3,9]. Despite these recommendations, 38% of Americans do not meet the Recommended Dietary Allowance (RDA) for calcium, and only half obtain the recommended intake through diet [10,11,12]. Even among patients within the osteoporosis care continuum who are identified and prescribed pharmacotherapy, calcium utilization remains below recommended goals. Two community pharmacy education interventions in patients prescribed osteoporosis therapy found that approximately half (45%, 55%, respectively) at baseline reported achieving 1000 mg of calcium supplementation [13,14]. Of the 28% of one study population taking proton pump inhibitors (PPIs), 75% were inappropriately taking them with calcium carbonate [13]. Therefore, care gaps also exist in the utilization of calcium and vitamin D.

Pharmacists in a variety of practice settings are uniquely positioned to optimize calcium and vitamin D in patients at risk from osteoporosis [6,7,13,14,15,16,17,18]. In a randomized controlled trial (RCT) of a community pharmacy education and quantitative heel ultrasound intervention for patients at risk for osteoporosis, there was a significant increase in the proportion of patients in the intervention group achieving a total daily calcium intake of 1500 mg [16]. Pharmacists included as part of multidisciplinary intervention in a long-term care setting significantly increased the proportion of residents prescribed calcium ≥ 500 mg/day and vitamin D ≥ 800 IU/day in the intervention group [17]. In a retrospective analysis of a pharmacist-run osteoporosis service in a family medicine clinic, patients significantly improved their intake of calcium (1200–1500 mg per day) and vitamin D (800–1000 IU) to goal [18]. Pharmacists also intervened if patients were inappropriately taking calcium carbonate with a PPI or Histamine2-receptor antagonist (H2RA) to remedy this medication-related problem (MRP).

While the literature supports the role of the pharmacist to optimize osteoporosis care, little research has been carried out to explore how to leverage specialty pharmacy services. In the US, specialty pharmacies are licensed pharmacies that provide complex medication therapy services to patients [19]. They are typically accredited by an independent third party through adherence to recognized quality practice standards [19]. Specialty pharmacists engage patients in comprehensive pharmacotherapy assessments, telephonic medication counseling and adherence monitoring in conjunction with the provision of prescription pharmacotherapy. As such, they may encounter patients at all points in the osteoporosis care continuum including–risk identification and screening, medication initiation, adherence optimization and lifestyle modification counseling. For patients receiving medications that reduce bone mineral density (BMD), the specialty pharmacist can identify and mitigate the risk through patient and prescriber interventions. For patients prescribed osteoporosis therapy, specialty pharmacists provide telephonic counseling on medications and lifestyle modifications along with adherence monitoring. This quality improvement project aimed to explore the impact of a semi-structured pharmacist-led intervention on patients’ daily calcium intake in a specialty pharmacy setting. We sought to improve calcium utilization by integrating comprehensive specialty pharmacy services, prescription and over-the-counter medication access and registered dietitian–nutritionist (RDN)-guided healthy foods choices within the grocery setting. The primary objective of this specialty pharmacy–RDN collaboration was to increase the percentage of patients achieving goal RDA calcium intake (1000–1200 mg) through a targeted intervention program.

## 2. Materials and Methods

### 2.1. Participants and Setting

The specialty pharmacy provides centralized processing for specialty medications within a chain of 77 community grocery store pharmacies. Patients receive comprehensive telephonic services including pharmacotherapy assessments and detailed medication counseling from specialty pharmacists. Patients then pick up their prescription medications directly from their community grocery store pharmacy providing ready access to over-the-counter medications and healthy food choices. Targeted patients were aged 18–90 years at-risk for osteoporosis due to receiving specialty prescription osteoporosis pharmacotherapy (denosumab, teriparatide, zoledronic acid) or specialty medications that reduce BMD (elagolix, oral prednisone > 5 mg/day for 3 months). Patients were identified as utilizing the index medications during the routine telephonic specialty pharmacy workflow by pharmacists and/or student pharmacist interns (in their fourth and final year of the professional pharmacy curriculum). This quality improvement project, designed to align patient care with recommended guidelines, was deemed by the Institutional Review Board at Albany College of Pharmacy and Health Sciences as not subject to IRB oversight (Protocol #24-015).

### 2.2. Patient Assessment

A telephonic patient encounter template was co-created by the pharmacists and RDN to gather necessary assessment data and provide standardized educational messaging. This tool was pilot tested prior to use and revised by the team (all authors, Supplementary Materials). In addition to pharmacists, three student pharmacists in their final experiential year of the curriculum were trained to complete the initial and follow-up encounters. To gather the data needed for the assessment component, pharmacists collected a dietary calcium intake, a detailed medication history, and the data required for the FRAX Fracture Risk Assessment Tool [20]. As guidelines recommend assessing dietary intake prior to implementing calcium supplements to avoid exceeding recommended intake, patients’ self-reported recall of dietary intake was obtained [2,3]. To estimate daily dietary calcium intake, an online calcium dietary assessment tool was selected by the pharmacy/RDN team that is readily available to practitioners and affords a practical way to swiftly complete a dietary assessment within the specialty pharmacy workflow [3,21]. Patient dietary preferences (e.g., vegan, lactose free) were also noted to customize nutritional counseling.

While it is standard specialty pharmacy procedure to collect a detailed medication history during workflow, specific focus was placed on assessing the administration technique patients utilized for calcium supplementation and medications with the potential for MRPs when combined with calcium (ex. PPI, H2RA or levothyroxine). Patients reported the salt form of calcium they utilize, the milligrams (mg) of elemental calcium it contained, how frequently they take it when taken, and if administered with or without food. Patients were asked to obtain their supplement bottle if they were unsure of any information. Having the bottle in hand also helped patients use the ‘Drug Facts’ label to specify the number of tablets that constitute the serving size needed to obtain the mg of elemental calcium. These details were needed to identify potential MRPs associated with calcium administration such as the inappropriate use of the carbonate salt in patients taking PPIs or H2RAs, administration of calcium carbonate on an empty stomach, exceeding the maximal amount of absorbable calcium per dose, or underdosing based on the labeling. Vitamin D supplement use was noted in a similar manner and patients were queried regarding obtainment of vitamin D levels in the prior year. Estimated milligrams of dietary calcium intake combined with supplement use yielded the total daily calcium intake. If patients were unable to provide this information at the time of the encounter, a follow-up encounter was scheduled within one week to attempt to garner the information needed.

The FRAX calculates fracture risk in postmenopausal women and men aged 40 to 90 years based on sociodemographic and health data [20]. Data collected to calculate the score included age, sex, weight, height, adult history of previous fracture, family history of hip fracture in patient’s mother or father, current tobacco smoking, current or prior glucocorticoid exposure (prednisolone ≥ 5 mg for more than 3 months), confirmed diagnosis of rheumatoid arthritis, secondary osteoporosis, and alcohol consumption (≥3 units/day). To include the BMD score in the FRAX assessment, the specific femoral neck BMD (in g/cm^2^) must be included in addition to the specific DXA scanning equipment used. If this information is not known, this field should be left blank as per the assessment’s instructions. As it was anticipated that most patients would not have this information and it is not readily accessible to specialty pharmacists, this field was left blank for all patients to ensure consistency. The FRAX score was not assessed for those 18–39 years of age.

### 2.3. Educational Intervention

At the conclusion of the assessment phase, patients received structured telephonic educational counseling from the pharmacists on their total calcium intake, how it compared to the RDA, and patient-specific recommendations to enhance dietary intake tailored to their preferences, as increasing dietary intake is the first line approach [3]. If patients were unable to obtain the recommended intake from diet, supplements were recommended, with calcium citrate recommended for those with reduced gastric acid, such as those on PPIs and H2RAs [3]. Patients were counseled on actual or potential MRPs and mechanisms to avoid them (e.g., separation of medication from the calcium supplement, changes to medication therapy, dividing calcium supplementation of >500 mg into two doses). Written patient-specific recommendations were documented on a standard letter template and mailed to patients along with calcium and vitamin D fact sheets (used with permission) selected by the team that defined goal RDA calcium and vitamin D intakes and identified calcium rich foods [10,21]. The mailing included in-store coupons for discounts on grocery items (cottage cheese, broccoli, fortified orange juice, soymilk, yogurt) selected by the RDN to reinforce calcium rich food selection.

### 2.4. Outcome Measures

Follow-up encounters were conducted by telephone 3–6 months after enrollment at a time aligned with the patient’s medication-refill date, to document any changes and to re-evaluate daily calcium intake. The primary endpoint was the proportion of patients who met the RDA for calcium (1000–1200 mg/day) at follow-up. Secondary outcomes included vitamin D utilization and identification, and resolution of MRPs related to calcium supplementation. Patient-reported care, including if the prescriber discussed calcium or vitamin D with the patient or assessed vitamin D levels, was collected. For patients taking index medications that reduce BMD, patients were asked if the prescriber discussed this risk with them. Redemption of in-store dietary-calcium food-source coupons were tracked on an aggregate (non-customer specific) basis for 6 months after final enrollment.

### 2.5. Statistical Analyses

Unadjusted descriptive statistics were used to summarize baseline characteristics and study outcomes. Continuous variables were reported as means with standard deviations, while categorical and dichotomous variables were summarized as counts and percentages. Two sets of analyses were conducted. First, the McNemar test was used to compare the proportion of patients meeting the RDA for calcium at follow-up versus baseline. In the primary outcome analysis, patients who were lost to follow-up had their baseline values carried forward. In a sensitivity analysis, these patients were alternatively classified as not meeting the RDA.

Second, analyses were conducted to identify baseline characteristics present in at least 10% of the study population that were associated with meeting the RDA for calcium at follow-up. Limiting the analysis to variables with a prevalence of ≥10% was intended to reduce the risk of overinterpretation. Categorical variables were compared using chi-square tests, with Fisher’s exact test applied when expected cell counts were <5. Continuous variables were compared using Student’s *t*-test. Baseline variables associated with meeting the RDA for calcium at a *p*-value < 0.10 in bivariate analysis were considered for inclusion in a multivariable logistic regression model. A backward stepwise elimination method was used to identify independent predictors of meeting the RDA for calcium at follow-up.

All analyses were conducted using IBM^®^ SPSS^®^ Statistics (version 30) for Windows. A *p*-value < 0.05 was considered statistically significant.

## 3. Results

Baseline characteristics of the study population are presented in Table 1. A total of 50 patients received the intervention, including 47 females and 3 males, with a mean age of 66.6 years (SD 15.3). As shown in Table 2, the average baseline daily dietary calcium intake was 500 mg (SD 247 mg), with 10% (*n* = 5) of patients consuming less than 200 mg per day. No patients met the recommended daily allowance (RDA) for calcium through diet alone. Calcium supplementation was reported by 22 patients (44%), with an average supplemental intake of 686 mg (SD 284 mg) per day (Table 2). As a result, the total daily calcium intake (dietary plus supplemental) averaged 811 mg (SD 461 mg), with no patient exceeding the established safe upper intake limit. Regarding vitamin D status, 30% (*n* = 15) of patients reported having a vitamin D level assessed within the past year; the remaining patients were either unsure or had not undergone testing. Of the 34 patients taking vitamin D supplements, only 13 reported vitamin D testing within the prior year. Notably, two patients reported taking 5000 IU of vitamin D daily—exceeding the safe upper limit—and both had undergone recent vitamin D level assessments. Patients who did not recall receiving vitamin D monitoring were advised to discuss this parameter with their health care providers.

Of the 50 patients enrolled, 34% (*n* = 17) met the RDA for calcium intake at baseline, compared to 60% (*n* = 30) following the intervention (*p* < 0.001). Nine patients were lost to follow-up and did not complete the follow-up assessment. Of these 9 patients, 8 had not met the RDA for calcium intake at baseline. When these 9 patients were conservatively classified as not meeting the RDA at follow-up, the results of the primary outcome remained unchanged. Among the 33 patients who were not at RDA for calcium intake at baseline, 13 (39.4%) met the RDA calcium intake goal at the follow-up visit.

A comparison of baseline characteristics between patients who met versus did not meet the RDA for calcium at follow-up is shown in Table 3. Baseline characteristics associated with meeting the RDA at a *p*-value < 0.10 included oral calcium supplementation, history of previous fracture, FRAX score, and FRAX hip score. In the multivariable logistic regression analysis, only oral calcium supplementation (adjusted odds ratio [aOR] 14.3; 95% confidence interval [CI]: 2.5–80.3) and a history of previous fracture (aOR 4.4; 95% CI: 1.01–20.0) remained independently associated with meeting the RDA for calcium at follow-up.

Regarding secondary outcomes, the intervention effectively identified and addressed MRPs related to calcium administration. All seven patients taking levothyroxine were appropriately separating their calcium intake—either from diet or supplements—by at least one hour. Among the six patients using calcium carbonate concomitantly with PPIs or H2RAs at baseline, three switched to calcium citrate to improve absorption at the time of follow-up. Additionally, three patients, initially misinterpreted the labeling on their calcium supplements and believing one tablet provided the mg noted on the label (vs. 2 tablets), were underdosing at baseline. These issues were corrected during follow-up, as confirmed by reassessment. In-store coupon utilization showed that more than half of the patients redeemed at least one coupon, with the most frequently selected items being cottage cheese (*n* = 27), broccoli (*n* = 18), orange juice (*n* = 12), soymilk (*n* = 6), and yogurt (*n* = 5).

## 4. Discussion

This specialty pharmacy quality improvement initiative increased patients’ daily calcium intake to the recommended goal. Our baseline findings, showing 34% of patients meeting the RDA of calcium intake, underscore the need for pharmacist intervention, even for those patients already in the care continuum prescribed osteoporosis pharmacotherapy. This low utilization at baseline was consistent with published national estimates and prior community pharmacy-driven educational interventions [3,11,13,14]. While filling this calcium gap, pharmacists are trained to counsel patients on potential MRPs associated with calcium ingestion. For instance, calcium can affect the absorption of certain medications necessitating appropriate timing of administration. Additionally, calcium carbonate absorption is diminished by PPIs, making calcium citrate a better option. Almost half of patients in this assessment were taking a PPI, with 25% taking the wrong calcium salt. Few studies detail the assessment of medication-related problems associated with calcium utilization [13,18]. The beginning phases of the development of a pharmacist-led osteoporosis intervention for optimizing medication use (PHORM) in primary care has been published with a focus on closing the osteoporosis care gap [22]. It is unclear how dietary and supplement interventions will be part of this large-scale pharmacist-primary care patient shared decision making model. Future research should evaluate on a larger scale the impact of pharmacists in osteoporosis medication optimization which includes calcium/vitamin D in addition to an assessment of medications, such as PPIs, known to have a negative impact on BMD in this at-risk population [2].

Patient understanding of supplement labels impacted appropriate dosing and administration. For example, while the front of the bottle indicated 500 mg of calcium, the drug facts label revealed the serving size was 2 or 3 tablets or gummies needed to obtain that amount, leading to patient confusion and underdosing prior to counseling. In contrast to previous studies, we did not identify patients consuming their total daily calcium supplementation in one dose rather than divided administration for optimal absorption of more than 500 mg [2,13,14]. Regardless, patient counseling should include dividing calcium supplementation of more than 500 mg per dose [2,3].

In addition to gaining a clearer understanding of calcium utilization, our intervention provided insight into patient–prescriber conversations. Only 38% of patients recalled discussing calcium and vitamin D intake with prescribers. When those prescribed elagolix (7) or chronic prednisone (5) were asked if prescribers reviewed the medications could reduce BMD, 2 stated yes, 4 stated no, and 6 were unsure. These findings may suggest either insufficient communication from prescribers and/or poor patient recall indicating the need for written materials to support verbal counseling. A number of patients stated a lack of vitamin D monitoring in the year prior to the intervention, indicating conversations surrounding follow-up monitoring and continued use of vitamin D may also be warranted to align with practice recommendations. These findings also highlight a gap in the care of patients at risk for osteoporosis due to prescribed medications that reduce BMD. While previous literature supports the role of the community pharmacist in reducing this risk, the role of the specialty pharmacist to mitigate this risk requires expanded study, as patients with medications that reduce BMD, particularly corticosteroid use in transplant and chronic inflammatory diseases, are frequently encountered in this setting [15].

Assessment of dietary intake is important to ensure patients do not exceed the calcium RDA and better understanding of patient preferences can encourage self-management. While utilizing patient dietary recall poses a potential limitation to the project, our baseline data were consistent with previously published national estimates of daily dietary intake [3,9,11]. To enhance reproducibility, we used a readily available tool that both pharmacists and patients can use, similar to that recommended to clinicians as a practical method for assessment [3,21]. Patient socioeconomic factors which could impact our primary outcome measure were not collected in this intervention. While a previous study of recommendations made by a fracture liaison service found that patient socioeconomic factors did not affect adherence to vitamin D supplementation, these factors cannot be overlooked when developing patient tailored recommendations [23]. The costs associated with calcium use and calcium-rich foods are not covered by most insurance; thus, the ability of patients to adhere to recommendations may be impacted by the patient’s ability to pay for or access them. While the literature continues to build on factors that influence patients’ decision making, pharmacists should continue to ascertain patient health beliefs, concerns, and socioeconomic factors to enable shared decisions to reduce the osteoporosis care gap [4].

There are several limitations of this quality assurance initiative. First, there was the selection of a process outcome (calcium use) as the measure of quality osteoporosis care vs. the use of a clinical endpoint (fracture, BMD). The evidence for calcium and vitamin D use is strong enough to warrant its incorporation into treatment guidelines and osteoporosis prescription medication trials; thus, we believed it was an important short-term process measure to assess [2,3,5,6,7]. The intervention’s single-arm, pre/post design, lack of a control group, and small sample size at a single center prevent clear attribution that observed improvements in calcium intake were due to the intervention of specialty pharmacists and/or small financial incentives. Other factors may have influenced patient behavior as patient participation in a control group can also yield behavior change to increase calcium intake [16]. The role of the specialty pharmacist to minimize the osteoporosis care gap warrants further large-scale, long-term study to determine if the behavior change can be sustained. When designing future osteoporosis care interventions related to calcium and vitamin D intake, researchers would need to balance the ethical considerations of a control group.

## 5. Conclusions

Calcium use among specialty pharmacy patients who are at risk for osteoporosis and/or receiving osteoporosis pharmacotherapy remains suboptimal. Specialty pharmacies can effectively identify patients with inadequate calcium intake and provide tailored educational interventions to improve intake levels and minimize potential MRPs. Specialty pharmacists embedded within grocery models can combine written and verbal communication alongside nutritional counseling and product provision to optimize specialty medication use. Future larger studies to identify scalable, replicable interventions within specialty pharmacies to narrow the osteoporosis care gap are warranted.

## Figures and Tables

**Table 1:** Patient demographics, medication use and osteoporosis risk factors (*n* = 50).

Mean (SD) age, years	66.6 (15.3)
Female sex	47 (94.0)
Race, Caucasian	40 (80.0)
Mean (SD) weight, kg	74.4 (20.0)
Mean (SD) height, m	1.6 (0.09)
Mean (SD) BMI, kg/m^2^	29.2 (8.0)
Previous history of fracture	20 (40.0)
Parental previous history of fracture	4 (8.0)
Current Smoking	5 (10.0)
Glucocorticoid use	6 (12.0)
Rheumatoid arthritis	6 (12.0)
Secondary osteoporosis	2 (4.0)
Alcohol use > 3 units per day	0
Mean (SD) FRAX 10-year probability major osteoporotic fracture **	17.9 (10.4)
Mean (SD) FRAX 10-year probability hip fracture **	6.6 (7.9)
Index medication for osteoporosis treatment	38 (76.0)
Denosumab	36 (72.0)
Teriparatide	1 (2.0)
Zolendronic acid	1 (2.0)
Index medication reduces BMD	12 (24.0)
Elagolix	7 (14.0)
Prednisone	5 (10.0)
PPI or H2RA Use	25 (50.0)
Levothyroxine Use	7 (14.0)

Data are presented as No. (%) unless otherwise indicated; ** 5 patients < 40 years of age not assessed with FRAX. Abbreviations: SD, standard deviation; BMI, Body Mass Index, BMD, Bone Mineral Density, PPI, proton pump inhibitor, H2RA, Histamine2-receptor antagonist.

**Table 2:** Baseline calcium and vitamin D care and daily intake (*n* = 50).

Patient Reported Care Received	Prescriber discussed calcium and vitamin D	19 (38)
	Vitamin D level assessed	15 (30)
	Prescriber discussed reduction in BMD associated with index medication (*n* = 12)	2 (16.7)
Calcium Intake	Mean Daily Dietary intake milligrams (SD)	500 (247)
	Patients using daily calcium supplement	22 (44)
	Mean Daily Supplement intake milligrams (SD)	686 (284)
	Mean Total Daily Calcium intake: dietary plus supplement use (SD)	811 (461)
	Patients meeting goal daily calcium intake	17 (34)
Vitamin D Intake	Patients using vitamin D supplement	34 (68)
	Mean Daily Supplement intake, units (SD)	1864 (1283)
	Patients meeting goal daily vitamin D intake	29 (58)

Data are presented as No. (%) unless otherwise indicated. Abbreviations: SD, standard deviation; BMD, Bone Mineral Density.

**Table 3:** Comparison of baseline characteristics present in ≥10% of the study population between patients meeting vs. not meeting the Recommended Dietary Allowance (RDA) for calcium at follow-up.

	Met RDA for Calcium Intake at Follow-Up Visit (*n* = 30)	Did not Meet RDA for Calcium Intake at Follow-Up Visit (*n* = 20)	*p*-Value
Mean (SD) age, years	68.4 (14.2)	64.2 (16.4)	0.17
Female sex	27 (90.0)	20 (100)	0.15
Race, Caucasian	25 (83.3)	15 (75.0)	0.47
Mean (SD) BMI, kg/m^2^	28.7 (8.1)	30.2 (7.9)	0.27
Index Medication: for osteoporosis treatment (*n* = 38)	24 (80.0)	14 (70.0%)	0.42
Index Medication: Denosumab	23 (76.7)	13 (65.0)	0.37
Index Medication Reduces BMD	6 (20.0)	6 (30.0)	0.42
Prescriber discussed calcium and vitamin D with patient	14 (46.7)	5 (25.0)	0.12
Calcium Oral Supplement Use	19 (63.3)	3 (15.0)	<0.001
VitaminD1 Oral Supplement Use	22 (73.3)	12 (60.0)	0.32
PPI or H2RA Use	14 (46.7)	11 (55.0)	0.56
Previous history of fracture	16 (53.3)	4 (23.5)	0.05
Mean (SD) FRAX 10-year probability major osteoporotic fracture **	20.8 (9.9)	12.8 (9.8)	0.005
Mean (SD) FRAX 10-year probability hip fracture **	8.2 (8.9)	4.0 (5.4)	0.04

Data are presented as No. (%) unless otherwise indicated; ** 5 patients < 40 years of age not assessed with FRAX. Abbreviations: SD, standard deviation; BMI, Body Mass Index, BMD, Bone Mineral Density, PPI, proton pump inhibitor, H2RA, Histamine2-receptor antagonist.

## Data Availability

The raw data supporting the conclusions of this article will be made available by the authors on request.

## Supplementary Materials

The following supporting information can be downloaded at: https://www.mdpi.com/article/10.3390/pharmacy13040100/s1, Calcium Project Encounter Template.
