# Supplementary material for: CalOPT: A Specialty Pharmacy–Dietitian Quality Improvement Initiative for Calcium Optimization in Patients with Osteoporosis Risk
Source: Pharmacy (Basel). 2025 Jul 23;13(4):100. doi: 10.3390/pharmacy13040100 (PMC12389519; doi:10.3390/pharmacy13040100)
Supplement: Supplementary file 1 [file pharmacy-13-00100-s001.zip › pharmacy-3631482-supplementary.pdf]

Calcium Project Encounter Template:

1. Is the patient on a medication for treatment of osteoporosis? Yes or No
   2.1 If yes, what medication: (check boxes: denosumab, teriparatide, zolendronic acid, romosozumab)

2. Is the patient on medication that increases the risk of osteoporosis? (Prednisone, Elagolix, Leuprolide Acetate, other : list)
3. 2.1 If yes, what medication: (check boxes: prednisone, elagolix, leuprolide acetate, other)
   2.2 Was the patient informed by a healthcare provider that this medication may cause bone loss? Yes/No

4. Medication Reconciliation completed and documented in pharmacy system:

5. Did your health care provider talk to you about getting calcium and vitamin D while on these medications?

To reduce the risk of bone loss or osteoporosis it is important to get enough calcium and vitamin D every day. We would like to gather some information about your diet and supplements to see how much you are getting and provide any recommendations to you.

6. Calcium from diet often comes in dairy products and green leafy vegetables. Tell me in a typical day how much of these you eat? { free text box}
   6.1 Ask patient to share what they ate yesterday and use online calcium calculator [https://www.osteoporosis.foundation/educational-hub/topic/calcium-calculator].
   6.2 Calculate estimated dietary calcium intake :_________________
   6.3 Is patient lactose intolerant/sensitive:
   - ❑ Yes
   - ❑ No

   6.4 Does the patient prefer to eat Vegan or have other dietary preferences:
   - ❑ Yes
   - ❑ No
7. Are you taking calcium supplements?
   - ❑ Yes
   - ❑ No

   7.1 If yes, Milligram amount:
   - ❑ Specify: ______
   - ❑ Unknown (generate a follow up intervention within 1 week to check back with patient set time prn)

   7.2 What salt form are you taking?
   - ❑ Calcium Carbonate
   - ❑ Calcium Citrate
   - ❑ Other (specify): ______
   - ❑ Unsure (generate a follow up intervention within 1 week to check back with patient set time prn)

   7.3 How often do you take the supplement?
   - ❑ Once daily
   - ❑ Twice daily
   - ❑ Three times daily

   7.4 Do you take the supplement with or without food?
   - ❑ With
   - ❑ Without

8. Are you taking a vitamin D supplement? Yes / No
   8.1 If yes: What type?
      6.2 How many units?
         - ❑ Specify: ______
         - ❑ Unsure (generate a follow up intervention within 1 week to check back with patient set time prn)

   8.3 Have you had recent bloodwork that included a vitamin D level?
      - ❑ Yes. If yes results?
         - Good Specify date checked: ______
         - Low Specify date checked: ______
         - Unsure
      - ❑ No
      - ❑ Unsure

9. Medication related problem with calcium supplements identified.
   - ❑ Drug interaction
      - Specify: ________
   - ❑ Administration problem / error
   - ❑ Wrong salt form
   - ❑ Other (specify): _______

10. FRAX Score - "To assess your future risk for factures, I would like to ask you a few more questions?" (patients between 40 and 90) https://www.sheffield.ac.uk/FRAX/tool.aspx?country=9

   10.1 Age / DOB (FRAX score for those between 40-90 years of age) Specify: ______
   10.2 Sex
   - ❑ Male
   - ❑ Female

   10.3 Weight Specify: _____ Pounds
   10.4 Height Specify: _____ inches
   10.5 Race Check boxes: (Caucasian, Black, Hispanic, Asian)
   10.6 Previous fracture?
   - ❑ Yes
   - ❑ No

   10.7 Parent family history (mother or father) fractured hip?
   - ❑ Yes
   - ❑ No/Unsure

   10.8 Smoking?
   - ❑ Current
   - ❑ Previous
   - ❑ Never

   10.9 Recent glucocorticoid use? (if currently exposed to oral glucocorticoids or has been exposed for more than 3 months at a dose of prednisolone 5mg daily or more (or equivalent doses of other oral glucocorticoids). Review patient medication reconciliation list and prescription profile.
   - ❑ Yes
   - ❑ No

   10.10 Have you been diagnosed by provider with rheumatoid arthritis?

- ❑ Yes
- ❑ No

10.11 Secondary osteoporosis? Includes type I DM, osteogenesis imperfecta, untreated long standing hyperthyroidism, hypogonadism, premature menopause (<45 years old), chronic malnutrition, chronic liver disease.

- ❑ Yes
- ❑ No

10.12 Do you consume alcoholic beverages?

- ❑ Yes If yes How much in a typical day? Specify: ____
- ❑ No

  Alcohol ingestion = 3 or more units ingested a day (1 Unit = standard glass of beer (285mL, 30mL of spirits, medium sized (120mL) glass of wine)

10.13 Final FRAX Score (specify): ________

11. Follow Up Monitoring
    10.1 Next Dr Appointment
    Specify:
    10.2 Blood Work
    Specify:

12. Review with patient with recommendations to increase calcium intake (Dietary/supplement) given patient information and preferences. Inform patient a packet will be arriving in the mail with these recommendations in addition to educational materials, their FRAX assessments, coupons and coversheet?

FOLLOW-UP CALL (3 to 6 MONTHS AFTER INITIAL ASSESSMENT pending rx)

1. Total daily calcium intake (add up dietary and supplemental):
   1.1 Since we talked last time, did you make any changes to your calcium or vitamin D intake? [text box to document changes]
   - ❑ Yes
     - ▪ Increase / Decrease
   - ❑ No
2. What changes did the patient make?
   - ❑ Bought more calcium rich foods (Specify: _______)
   - ❑ Began using a supplement Calcium supplement
     - ▪ How did you take your supplement (dose formulation, mg, frequency)
       - Specify: ________
   - ❑ Began using a vitamin D supplement
   - ❑ Changed how they were using a supplement (Specify: ____)
   - ❑ Other (specify: ________)
3. Medication assessment
   3.1 How do you feel like the medication is working for you? (Specify: _______)
   3.2 Have you experienced any adverse effects from this medication?
   - ❑ Yes (specify: ______)
   - ❑ No

   3.3 Review if there were any medication related problems from baseline, and if resolved?
   - ❑ Specify: _________

   3.4 Have you started any new medications in the past 3 months?
4. Have you had any falls in the past 3 months?
   - ❑ Yes (specify date/any details: ______)
   - ❑ No
5. Have you had any fractures in the past 3 months?
   - ❑ Yes (specify date/any details: _____)
   - ❑ No
6. Document any pertinent lab information shared by patient
   - ❑ Specify: _________
7. Document any pertinent MD appointment information
   - ❑ Specify: _________
8. Free note area for any other questions, comments, concerns, etc
